# Clinical Significance of Telomere Length and Associated Proteins in Oral Cancer

**Published:** 2007-02-14

**Authors:** Rachana N. Sainger, Shaila D. Telang, Shilin N. Shukla, Prabhudas S. Patel

**Affiliations:** 1 Biochemistry Research Division, The Gujarat Cancer & Research Institute, Asarwa, Ahmedabad–380 016, India; 2 Department of Biochemistry, M.S. University of Baroda, Vadodara–390 001, India; 3 Deputy Director, The Gujarat Cancer & Research Institute, Asarwa, Ahmedabad–380 016, India

**Keywords:** Telomere, TRF-1, TRF-2, prognosis, oral cancer

## Abstract

**Purpose:**

Telomere shortening is an important event during carcinogenesis. Although studies suggest role of multiple proteins in telomere length regulation, there is dearth of reports in oral cancer which is a leading malignancy in Asian countries especially in India. Thus the present study was carried out to study these mechanisms and explore the pathways involved in telomere—telomerase regulation and identify possible prognostic markers to understand the biology of oral tumors for better treatment approaches.

**Methods:**

Telomere length was determined by Southern Hybridisation method, telomeric repeat binding factor (TRF) 1 and 2 expression was detected by Western blot method and telomerase activation by telomeric repeat amplification protocol. Statistical analysis was done using SPSS (Version 10) software.

**Results:**

Significant shortening of telomeres was seen in the tumor tissues as compared to normal tissues. Poor prognosis was observed in the patients with higher telomere length in malignant tissue, higher tumor to normal telomere length ratio (T/N TRF LR). Expression of TRF-2 but not TRF-1 protein was significantly higher in the malignant tissues. We also observed telomerase activation in 75 malignant tissues.

**Conclusions:**

Our results reveal significant clinical usefulness of telomere length, T/N TRF LR and telomerase activation in the prognosis of oral cancer patients. TRF-2 overexpression in malignant tissues appears to play an important role in telomere length shortening in oral cancer.

## Introduction

Telomeres are the extreme ends of double stranded eukaryotic chromosomes comprising tandem array of TTAGGG repeats and DNA binding proteins. Telomeric sequences vary among different organisms but are usually composed of long arrays of guanine rich sequences (Blackburn, 1991). Human telomeres vary with age and cell types and in general range from 6 to 12 kb length in the somatic cells. In humans, it consists of repeats of TTAGGG with a 3’ end overhang that helps in the formation of D-loop and T-loop structures. Telomeres protect the chromosomal ends from degradation by exonucleases; prevent recognition as double stranded DNA breaks, end-to-end fusions, and ring chromosome formation. Thus, telomeres play a vital role in the regulation of gene expression, functional organization of the chromosome and in controlling the replicative life of cells and entry into senescence.

Recently, it is demonstrated that telomere attrition is an early event in the epithelial carcinogenesis (Meeker et al. 2004). Different proteins, like telomeric repeat binding factors (TRF-1 and TRF-2), bind to the double stranded telomeric DNA and help in regulating telomere length by different mechanisms, either as inhibitors of telomerase or activators of telomere degradation. TRF-1 is expressed ubiquitously throughout the cell cycle and binds to TTAGGG repeat as a homodimer with great specificity. It inhibits telomerase-dependent elongation and participates in regulation of the mitotic spindle. TRF-1 is regulated by other proteins and acts as negative regulator of telomere length (telomerase-dependent pathway). On the other hand, TRF-2 stabilizes the G-rich strand overhang and inhibits telomere-telomere fusions. TRF-2-negative telomeres are recognized as damaged DNA. Over expression of TRF-2 in somatic cells leads to shortening of telomeres thus acting as a negative regulator of telomere length. TRF-2 inhibition is reported to cause apoptosis and non-homologous end joining of telomeres. The knowledge about proteins that bind to the single stranded and double stranded telomeric repeats has increased in recent years (Oh et al. 2005; Yamada et al. 2002; Fujimoto et al. 2003), however; there are very few reports showing the expression status during carcinogenesis. Although TRF-1 and TRF-2 expression is reported in hemopoeitic cells (Yamada et al. 2000), adrenal tumors (Kanauchi et al. 2003), lung tumors (Nakanishi et al. 2003) and pancreatic tumors (Yajima et al. 2001), there are no reports in oral cancer.

Earlier work from our laboratory suggested significant clinical usefulness of telomere length analysis and telomerase activation in head & neck tumors (Patel et al. 2002). We have also observed that telomerase activation, telomerase reverse transcriptase (hTERT) expression are predictors of poor survival in the oral cancer patients (*Unpublished data*). Considering these, the present study was carried out to understand the mechanisms involved in the regulation of telomere length in oral cancer, a leading malignancy in India. The study was designed to determine the telomere length changes, telomerase activation and expression of TRF-1 and TRF-2 in oral tumors and to evaluate their clinical significance. Tumor to Normal peak TRF Length Ratio (T/N TRF LR) was also calculated to establish its clinical significance in oral cancer.

## Materials and Methods

### Patients and samples

Hundred untreated oral cancer patients were enrolled from the Out Patients department of the Gujarat Cancer & Research Institute. The diagnosis was established by clinical, histopathological and radiological investigations. Major site of cancer was buccal mucosa (n = 36) and tongue (n = 28) and others including alveolus, GB sulcus etc. Mean age of the patients was 45 years (Age range: 25–72 years). Tumor staging was done by TNM classification of tumors (AJCC, 1997) which is based on the tumor size, lymph node involvement and metastasis of the tumor. Histopathologically, the tumors represented squamous cell carcinoma. 80% cases were from stage advanced disease (III and IV) and lymph node metastasis was evident in 44% oral cancer patients. Tissues specimens from the patients were collected at the time of surgery. Tumor tissue and adjacent normal tissues were selected by a pathologist and later confirmed by the histopathological examination. Adjacent normal tissues were taken from tissue 1 cm or more away away from the tumor margins whereever possible and were defined as pathologically normal and were used to compare with the tumor tissues.

### Telomere length by Southern Hybridization method

Telomere length changes in the tissues were determined by Southern hybridization method (Harley et al. 1990). DNA was extracted from the tissues using standard DNA extraction protocols and digested using Hinf1 enzyme. Digested DNA samples were run on 0.7% agarose gels, transferred using capillary transfer method and the membranes were hybridized with Digoxigenin (DIG) labeled telomeric probe (TTAGGG)n. Signals were detected using DIG Chemiluminescence detection method and images were captured using Gel documentation system, (BioRad, USA). Telomere length was measured as peak Terminal Restriction Fragment length (TRF), represented in kilo base pairs (kbp), which corresponds to the highest peak obtained in densitometric scan (Sashida et al. 2003). To avoid discrepancy, the range of peak TRF length observed in the tissue and mean values are also provided. The Peak TRF length was distributed into two groups, above and below median for the survival analysis. We also calculated the ratio of tumor to normal TRF length (paired tissues) and grouped as above and below median values.

### Telomerase activation by TRAP assay

Telomerase activation was analysed by standard Telomeric Repeat Amplification Protocol (TRAP) using commercially available kit (Roche Molecular Biochemicals, Germany). Malignant and adjacent normal tissues were pulverized using liquid nitrogen, to this 200 μl of ice cold lysis reagent was added for cell lysis and incubated on ice for 30 minutes. Lysate were centrifuged for 20 minutes at 15,000 rpm at 2–8°C. The supernatant was collected in a fresh tube. Protein concentration was estimated by Folin Lowry method (Lowry et al. 1951). BSA was used as a standard for the calculation of proteins. Aliquots of the supernatant were prepared and stored at − 80°C, until analyzed. 50 μg protein aliquot was used for the telomerase mediated primer elongation and later amplified by PCR and the products were run on 15% denaturing PAGE, transferred onto nylon membrane detected by Chemiluminescence detection kit (Roche Molecular Biochemicals, Germany). 293 kidney cells provided with the kits were used as telomerase positive control and lysis buffer was used for negative control. The samples, which showed characteristic six base pair DNA ladder, were considered telomerase positive.

### Expression of TRF-1 and TRF-2 by Western blot method

Tissues were homogenized in phosphate buffer saline (pH: 7.4) and centrifuged at 15,000 rpm for 15 minutes. Supernatant were collected and used for determination of protein content by Lowry method. Aliquot equivalent to 100 μg protein was used for electrophoresis. Transfer of protein to ECL membrane was done by Semi Dry transfer method Multiphore II Nova Blot Unit (Amersham Biosciences, U.S.A.). Monoclonal antibodies against TRF-1 and TRF-2 (Calbiochem, U.S.A.) were used at a concentration of 1 μg/ml and secondary antibody was diluted 1:1000 in Tris buffered saline. Detection was carried out by ECL detection kit (Amersham Biosciences, U.S.A.). Scanning and quantitation of bands was carried out by Gel Documentation system (Bio Rad, U.S.A.) and results were represented as OD/mm^2^.

### Statistical analysis

Statistical analysis was carried out using SPSS software (Version 10). Comparison of means was done by unpaired and paired t-tests. ANOVA and multivariate tests were performed to determine the association of markers with clinical parameters. Kaplan Meier survival curves were plotted to determine the effect of peak TRF length, T/N TRF LR on the survival of patients. The comparison of the survival in the two groups was done by Log Rank Statistics. For the statistical analysis p value <0.05 was considered significant.

## Results

### Peak TRF length in the tissues and association with clinical details

**Figure 1:**
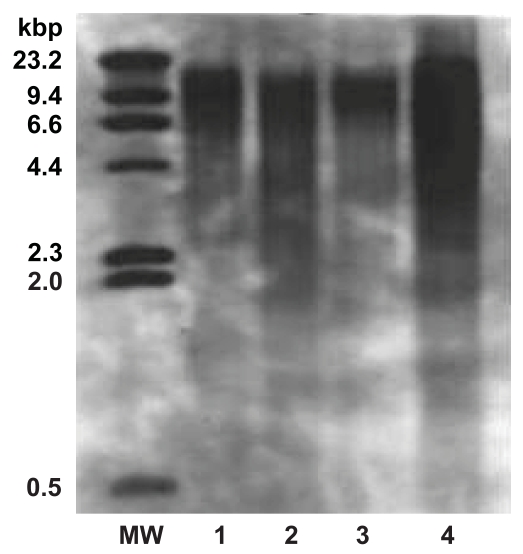
**Representative pattern for peak TRF length in tissues.** MW: Represents the DIG labeled DNA molecular weight marker (0.5–23.2 kilo base pairs). Lane 1 & 2: Peak TRF length in adjacent normal and malignant tissue specimens from patient with carcinoma of buccal mucosa. Lane 3 & 4: Peak TRF length in adjacent normal and malignant tissues from patients with carcinoma of tongue.

In the present study peak TRF length ranged from 4.1 to 15.6 kbp in the malignant tissues and 3.8 to 17.6 kbp in the adjacent normal tissues as shown in Table 1. Representative pattern for peak TRF length in the malignant and adjacent normal tissues of patient with carcinoma of buccal mucosa and tongue is given in Figure 1. Students t-test (unpaired and paired) demonstrated that mean peak TRF length in the malignant tissues (8.18 kbp) was significantly shorter (p < 0.001) then the adjacent normal tissues (9.97 kbp).

We also observed that increase in peak TRF length was associated with progression of disease, well differentiated tumors as well as increasing tumor size (Table 2). Also peak TRF length in the lymph node positive patients was shorter then the lymph node negative patients. A decrease in telomere length was observed with increasing age which was statistically not significant. Further, in multivariate analysis to determine the association of telomere length changes, we found a significant association between tumor differentiation and telomere length (p = 0.05) only (Table 3).

### Survival analysis

**Figure 2:**
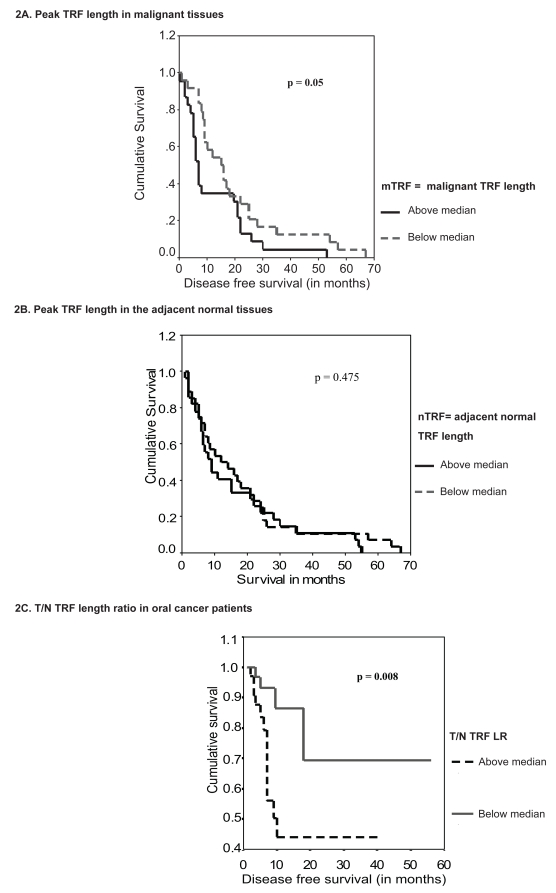
Survival curves for peak TRF length and T/N TRF LR in oral cancer patients.

Effect of telomere length on survival of the patients was determined by Kaplan Meier survival method. For this median peak TRF length for the malignant and adjacent normal tissues were determined and the patients were grouped into above and below median groups. Survival analysis revealed that patients having higher peak TRF length in the malignant tissues had significantly poor five-year disease free survival as compared to the patients showing peak TRF length less than the median (Figure 2A). Thus, it is evident that higher telomere length might be indicative of increased capability of the cells to undergo additional number of divisions, hence longer proliferating capacity.

### T/N TRF LR in the patients

The ratio of tumor and respective adjacent normal TRF length to nullify the telomere length differences between two groups were determined. The ratio ranged from 0.28 to 1.55 in the patients and was grouped into above and below median group. Although T/N TRF LR was not associated with the clinical features of tumor, it showed significant association with the survival of the patients (Figure 2B). Patients having higher T/N TRF ratios showed significantly shorter survival of 21.2 months as compared to the patients having lower ratio who had survival of 42.8 months. The present results suggest that higher T/N TRF LR in patients may confer growth advantage to the tumors by providing longer proliferating capabilities.

### Expression of TRF-1 and TRF-2 and association with clinical parameters

**Figure 3:**
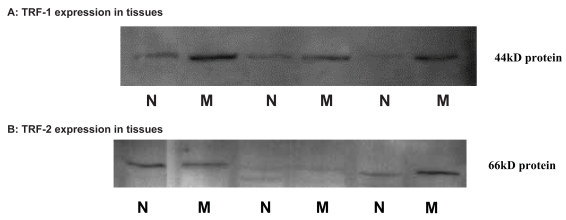
**Representative pattern for expression of TRF-1 and TRF-2 in the malignant and adjacent normal tissues.** M = Malignant tissues. N = Adjacent normal tissues.

Figure 3 exhibits representative pattern for expression of telomeric repeat binding proteins carried by Western blot method. Expression of TRF-1 protein was detected in 56.0% malignant and 20.0% adjacent normal tissues while TRF-2 expression was seen in 76.0% malignant and 44.0% of the adjacent normal tissues. When the mean expression levels were compared (Table 4), TRF-2 protein expression was significantly higher in the malignant tissues (p < 0.001) then the adjacent normal tissues.

One-way ANOVA was carried out to determine the association of TRF-1 and TRF-2 expression with the clinicopathological factors. ANOVA analysis revealed inverse relation of TRF-1 expression with disease stage, tumor differentiation and tumor size however no association was observed with lymph node metastasis. None of the differences were statistically significant in the present analysis. In the adjacent normal tissues, TRF-1 expression was significantly associated with the disease progression i.e. from stage 1 to stage 4. Details of ANOVA test are provided in Table 5A. Further, in the adjacent normal tissues TRF-1 protein levels showed significant association with increasing tumor size. This might be suggestive of role of TRF-1 expression during the clinical course of disease. Expression of TRF-2 protein was significantly associated with the disease stage in the adjacent normal tissues. In multivariate analysis, we did not observes association between TRF-2 expression and clinicopathological features like stage, tumor differentiation, nuclear grade, tumor size and nodal invasion was observed as shown in Table-6.

### Telomerase activation in tissues and association with telomere length

**Figure 4:**
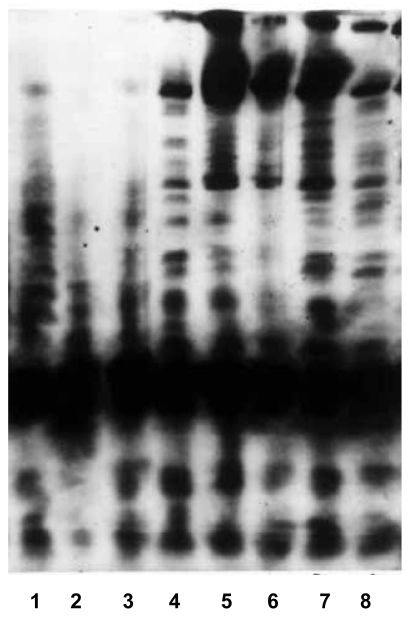
**Telomerase activation in the tissues.** N = Adjacent normal tissue; M = Malignant tissue; +ve = positive control; –ve = negative control. Lane-1 and lane-2 represent telomerase positive control, 293-kidney cell lysate and telomerase negative control respectively. Lane 3, 4 represents telomerase negative adjacent normal tissue and telomerase positive malignant tissue of buccal mucosa patient. Lane 5, 6 represent telomerase positive adjacent normal and malignant tissue from tongue carcinoma patient. Lane 7, 8 represents telomerase positive adjacent normal and malignant tissues from buccal mucosa patient.

**Figure 5:**
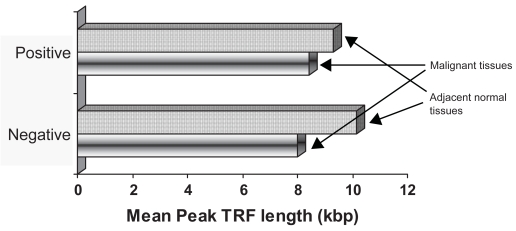
**Mean Peak TRF length in the telomerase positive and negative tissues.** Telomerase positive tissues had shorter telomere length then the telomerase negative tissues specimens.

A characteristic six base DNA ladder in the specimens was considered to be telomerase positive (Figure 4). Telomerase activation was observed in 75% of the tumor tissues and 62.4% of the adjacent normal tissues. Further, peak TRF telomere length in the telomerase positive and negative tissues was compared. Normal tissues in both telomerase positive and negative tissues showed higher telomere length then the tumor tissues (Figure 5).

### Correlation of proteins with telomere shortening

Spearman’s correlation coefficients were computed to determine association of proteins with telomere length. TRF-1, TRF-2 expression and telomerase activation showed a negative association with telomere length changes in the tissues (p = 0.30; 0.79; 0.17 respectively).

## Discussion

Telomeres are progressively shortened at each cell cycle due to end replication problem faced by the eukaryotic cells which finally leads to the growth arrest of these cells at a stage termed as senescence. However, cells acquire additionally forced proliferation capacity which finally leads to significant telomere shortening (Wright and Shay, 2005). It is known that loss of capping function of telomeres results in chromosomal end to end fusion and formation of ring chromosomes (van Steensel et al. 1998). Also, telomeres when not replicated are bound by protein complex and protected by degradation (Wright and Shay, 2005).

In the present study, the peak TRF length in the malignant tissues ranged from 4.1 to 15.6 kbp while in the adjacent normal tissues it varied from 3.8 to 17.6 kbp. We found significantly shorter peak TRF length in the tumor tissue as compared to their normal counterparts in paired t-test, which suggests that telomere shortening occurs during carcingenesis. Other investigators (Lantuejoul et al. 2005; Gertler et al. 2004; Plentz et al. 2004; Oh et al. 2005; Patel et al. 2002) have also reported shorter telomere length in the tumor tissues. Recently Meeker et al. (2005) also reported that telomere length abnormalities are one of the earliest genetic alterations during the multi step epithelial carcinogenesis.

We observed inverse association between telomere length and lymph node metastasis. Telomere length alterations also showed negative association with age and tumor differentiation in the adjacent normal tissues which was not evident in tumor tissues. Similar association has been reported by Wu et al. (2003) in multiple myeloma patients. Telomere length is significantly associated with advanced disease (Gertler et al. 2004), tumor pathology and grade (Plentz et al. 2004).

Survival analysis suggested significantly shorter survival in the patients having longer telomere length in the malignant tissues than those with shorter telomere length. This may be because the cells need to proliferate continuously for longer duration for the survival of tumors. Likewise, it is true that cells with longer telomeres will survive for more cell divisions and will have longer life span, thus conferring poor disease free survival among the patients. Earlier similar association was reported in head & neck cancer (Patel et al. 2002), endometrial tumors (Menon et al. 2003), and colorectal carcinoma (Rosenberg et al. 2003). This observation was further demonstrated by determining T/N TRF LR. Higher T/N TRF ratio is reported to be significant indicator of poor survival in lung and gastric cancers (Hsu et al. 2004). In our study group we found significant association of higher T/N TRF LR with shorter survival of oral cancer patients. The T/N TRF LR takes into account the differences in the tissue pairs as well as the cellular ageing related changes. Thus, telomere length changes measured in terms of T/N TRF ratio is a better marker to determine the actual significance of telomere length alterations during cancer.

The double-stranded TTAGGG repeats of mammals are bound by two related proteins, TRF-1 and TRF-2 (Chong et al. 1995; Broccoli et al. 1997). Overexpression of TRF-1 results in telomere shortening, whereas mutants that are defective in DNA binding give rise to telomere lengthening (Smogorzewska et al. 2000). If TRF-2 is removed from telomeres in human cell lines the chromosome ends are immediately recognized as sites of DNA breaks, leading to chromosome end-to-end fusions, the activation of the p53 and p16/RB pathways, and the induction of senescence or apoptosis (Smogorzewska and de Lange, 2004). The expression levels of both TRF-1 and TRF-2 were higher in the malignant tissues as compared to their normal counterparts. So far, there are no studies on the role of TRF-1 and TRF-2 proteins in human oral cancer. In human oral cancer cell lines, Fujimoto et al. (2003) have shown involvement of TRF-1 up-regulation in normal cell senescence. Earlier studies have presented controversial data for these telomeric genes in cancer. Some studies suggested that TRF-1 and TRF-2 were down-regulated in tumor tissues (Yamada et al. 2002; Saito et al. 2002; Yamada et al. 2000), whereas others showed that TRF-1 or TRF-2 was up-regulated (Oh et al. 2005; Nakanishi et al. 2003; Ancelin et al. 2002). It is also suggested that in the absence of TRF-1, TRF-2 takes over the function of stabilizing telomeres along with the telomere interacting proteins and thereby block the access to telomerase (Smogorzewska and de Lange, 2004).

Loss of TRF-1 can result in telomere lengthening and extended life span of cells. Expression of TRF-1 is considered to be a homeostatic mechanism to control the proliferative potential of normal cells and directly acts by inhibiting telomerase activity (Smogorzewska et al. 2000). Overexpression of TRF-1 is associated with decreased telomere length via inhibition of telomerase activity in the cells. One possible mechanism to explain the role of TRF-1 in telomere maintenance suggested sequential post-translational modifications of TRF-1 which regulated access of telomerase to telomeres (Chang et al. 2003). Normally, TRF-1 is in configuration that blocks access to telomerase, however tankyrase ribosylates TRF-1 and removes them from telomeres. TRF-1 complex removed from telomeres is then degraded by proteosome complex. Telomerase causes extension of telomeres. Once the telomeres are lengthened, newly synthesized TRF-1 reassemble and form a telomerase inaccessible state. The constant shuttling of TRF-1 helps in the maintenance of telomere length.

Inverse association of TRF-1 expression with disease stage, tumor differentiation and tumor size was observed. In the adjacent normal tissues, TRF-1 expression was significantly decreased with increase in disease stage. This is possibly due to the fact that in advanced disease requirement of TRF-1 and TRF-2 molecules is reduced, due to shorter telomere length in the tissues. In breast cancer TRF-1 expression was associated with tumor size (Saito et al. 2002). Association of telomeric proteins, TRF-1 and TRF-2, appears to be different in various malignancies and needs to be clearly defined. In oral cancers, our data suggests that TRF-2 over expression seems to be an important event for telomere length regulation, however further studies in oral cancer cells and human tissues will enable to asses the potential use of these proteins.

Telomerase activation is observed in more than 80% of cancers but is absent in most of the normal somatic cells. Telomerase activation is required to prevent further loss of telomeres and maintain telomere length. In previous study (Sotillo et al. 2004) shorter telomere length was observed in the patients with detectable telomerase activity (p = 0.041), however we did not observe any difference in telomere length in telomerase positive and negative groups. This might be due to certain telomerase independent pathways like alternate lengthening of telomeres (ALT pathway) which exist in the cells (Muntoni and Reddel, 2005). Usually ALT positive cells demonstrate longer telomeres as compared to ALT negative cells. Thus apart from telomerase, several other proteins appear to be an important integral requirement for the maintenance of telomere length.

The study deciphers important clues regarding telomere biology in oral tumors, a malignancy less explored with this aspect. Telomere length and T/N TRF LR prove to be useful tools to determine the prognosis of oral cancer patients. Over expression of TRF-2 protein in the oral tissues suggests loss of capping function that results in end to end fusion often observed in cancer cells. Thus, it reflects that telomere dependent genomic alterations caused due to imbalance of proteins at the telomeric end play a major role in cancer and might help in identifying newer therapeutic targets. At the time when telomere and telomerase are being considered as a potential drug targets, it becomes imperative to identify the role of these proteins in both cell culture studies and human studies. Further analysis of these proteins is essential to strengthen the interesting observations of the present work and elucidate the mechanism involved in telomere length shortening in oral cancer.

## Figures and Tables

**Table 1:** Mean Peak TRF length (in kbp) in the tissues.

Tissues (N = 85)	Range of peak TRF length (in kbp)	Mean peak TRF length (in kbp)
Malignant tissues	4.1 to 15.6	8.18
Adjacent normal tissues	3.8 to 17.6	9.97

Student t-test and paired t-test: p value < 0.001

**Table 2:** Association of peak TRF length with the clinicopathological features of tumors.

Peak TRF length →(in kbp)	Malignant tissue (N = 85)	Adjacent normal tissue (N = 85)
**Age groups**		
**I**	8.18	9.6
**II**	7.87	10.11
**III**	8.60	10.4
**Tumor differentiation**		
Well	7.7	10.23
Moderate	8.35	9.53
Poor	9.51	10.1
**Disease Stage**		
Stage 1	6.75	7.34
Stage 2	7.72	9.27
Stage 3	10.1	10.01
Stage 4	8.28	9.35
**Nuclear grade**		
I	7.47	10.04
II	8.42	9.32
III	9.13	10.75
**Tumor size**		
T1	8.11	8.23
T2	8.08	9.27
T3	9.30	10.30
T4	7.94	10.44
**Lymph node status**		
Positive	8.0	9.23
Negative	8.45	10.38

**Table 3:** Multivariate analysis for association of peak TRF length with clinicopathological features of tumors.

	Malignant tissue		Adjacent normal tissue	
Parameters	F	‘p’ value	F	‘p’ value
Age	0.923	0.58	0.893	0.61
Sex	1.949	0.17	1.358	0.25
Differentiation	**2.935**	**0.05**	1.906	0.15
Nuclear Grade	2.202	0.13	0.543	0.58
Lymph Node	0.586	0.45	1.499	0.23
Stage	0.019	0.89	0.507	0.48
Habit	0.220	0.88	0.257	0.85

**Table 4:** Mean values of TRF-1 and TRF-2 in the tissues.

Tissues(N = 25)	Mean ± S.E.	Significance
**TRF-1**		
Malignant	1.61 ± 0.52	**0.49^NS^**
Adjacent Normal	1.05 ± 0.62	
**TRF-2**		
Malignant	9.54 ± 2.58	0.005*
Adjacent Normal	2.03 ± 0.86	

**Table 5A:** ANOVA table for association of TRF-1 with clinical factors.

	Malignant tissue		Adjacent normal tissue	
TRF-1 Factor	F value	‘p’ value	F value	‘p’ value
Stage	1.084	0.380	9.900	**<0.01**
Differentiation	0.936	0.443	1.211	0.333
Nuclear grade	1.530	0.240	2.100	0.147
Tumor size	0.624	0.609	6.900	**0.003**
Nodal invasion	0.536	0.472	0.348	0.562

**Table 5B:** ANOVA table for association of TRF-2 with clinical factors.

	Malignant		Adjacent Normal tissue	
TRF-2 Factor	F value	‘p’ value	F value	‘p’ value
Stage	0.133	0.939	5.250	**0.010**
Differentiation	0.098	0.960	0.080	0.770
Nuclear grade	0.088	0.916	0.119	0.885
Tumor size	0.599	0.626	1.659	0.218
Nodal invasion	0.083	0.777	0.326	0.575

**Table 6:** Multivariate analysis for association with TRF-1 and TRF-2 expression in the tissues.

Parameters	TRF-1 expression		TRF-2 expression	

	‘F’ value	Significance	‘F’ value	Significance
Age	0.701	0.695	0.859	0.618
Nuclear Grade	1.495	0.281	0.290	0.756
Lymph Node	0.841	0.386	0.409	0.541
Stage	0.081	0.923	0.106	0.901

## Abbreviations

TRF: Terminal restriction fragment
TRF-1: telomeric repeat binding factor-1
TRF-2: telomeric repeat binding factor-2
T/N TRF LR: Tumor/Normal TRF length ratio
